# Effects of recreational SCUBA diving practiced once a week on neurohormonal response and myokines-mediated communication between muscles and the brain

**DOI:** 10.3389/fcvm.2023.1074061

**Published:** 2023-03-29

**Authors:** Marina Njire Braticevic, Marko Zarak, Brankica Simac, Antonija Perovic, Jerka Dumic

**Affiliations:** ^1^ Department of Laboratory Diagnostics, Dubrovnik General Hospital, Dubrovnik, Croatia; ^2^ Clinical Department for Laboratory Diagnostics, Dubrava University Hospital, Zagreb, Croatia; ^3^ Faculty of Pharmacy and Biochemistry, University of Zagreb, Zagreb, Croatia; ^4^ Department of Biochemistry and Molecular Biology, Faculty of Pharmacy and Biochemistry, University of Zagreb, Zagreb, Croatia

**Keywords:** recreational scuba diving, myokines, muscle-brain crosstalk, pituitary-target organ hormones, brain damage markers

## Abstract

**Objective:**

During physical activity, activation of muscular, endocrine, and nervous systems, results in intensive crosstalk between muscles and other organs, which enables response to physiological stress. In SCUBA diving, extreme environmental conditions represent an additional challenge for homeostasis maintenance, but underlying mechanisms are largely unknown. We aimed to contribute to the understanding of neurohormonal response and muscle-brain crosstalk by measuring the concentrations of the selected hormones secreted by the pituitary-target organ axis and myokines involved in the muscle-brain endocrine loop in recreational SCUBA (rSCUBA) divers.

**Methods:**

Fourteen male divers performed five open-water recreational dives (one per week, depth of 20–30 m, lasting 30 min, between 9 and 10 am), after a winter non-diving period of 5 months. Blood samples were collected immediately before and after the first, third, and fifth dives. Adrenocorticotropic hormone (ACTH), cortisol, thyroid-stimulating hormone (TSH), free thyroxine (fT4), prolactin, total testosterone, growth hormone (GH), insulin-like growth factor-1 (IGF-1), irisin, brain-derived neurotrophic factor (BDNF), S100B, glial fibrillary acidic protein (GFAP), and neuron-specific enolase (NSE) were measured using commercially available immunoassays.

**Results:**

Cortisol and ACTH levels decreased after every dive, while total testosterone decreased only after the first dive. No significant changes in post-dive values, as well as the cumulative effect on any other measured hormone, were observed. Although irisin and BDNF levels decreased after the first and third dives, the fifth dive caused a significant increase in both myokines. Changes in IGF-1 levels were not observed. All three dives caused a significant increase in S100B levels. A statistically significant decrease in GFAP concentration was observed after every dive, while NSE pre-dive concentration declined over the studied period. The cumulative effect on myokine levels was reflected in a continuous decline in irisin and BDNF pre-dive levels throughout the studied period, but an increasing trend after the fifth dive was observed.

**Conclusions:**

Observed changes in myokines and hormone levels point to a specific response to rSCUBA practiced once a week, most likely due to extreme environmental conditions. Further studies on communication between muscles and other organ systems, particularly on the muscle-brain endocrine loop, are required for a deeper understanding of the adaptation mechanisms to this kind of physiological stress.

## Introduction

Physical exercise is essential for maintaining and improving health as well as reducing the risk of developing various diseases such as aging-related diseases, metabolic and cardiovascular diseases as well as cancer (1). Other long-term health benefits of physical activity include the induction of structural and functional changes in the brain, influencing cognition and well-being (2). However, some physical activities may have negative effects on health, depending on intensity, duration, and specific environmental conditions. Recreational SCUBA (self-contained underwater breathing apparatus) (rSCUBA) diving is a widespread, popular sport, with an increasing number of new athletes every year. The frequency of rSCUBA dives varies a lot, but it is usually practiced daily or even twice a day, during a short vacation period. Yet, in coastal countries, local rSCUBA divers usually practice it weekly, representing their continuous physical activity over the year, except during the winter non-dive period of a few months. Negative effects due to residual nitrogen in repetitive dives (two consecutive dives) have been recognized and safety recommendation has been published (3). However, the cumulative effect of rSCUBA diving practiced once a week has not yet been studied.

According to the Universal standards and procedures of the World Underwater Federation, SCUBA diving is a form of diving up to a depth of 40 m with direct access to the surface (without decompression stop) using compressed air or nitrox (a mixture of oxygen and nitrogen where the oxygen content does not exceed 40%) as a breathing gas (4). Due to increased ambient pressure, immersion, exposure to cold water, hyperoxia, and breathing at high-pressure underwater, it is considered a form of moderate-intensity exercise in extreme environmental conditions. To maintain homeostasis, the human organism activates numerous processes that lead to functional adaptations of many organ systems during and after the dive (5, 6).

At the onset of exercise, as the body starts to move, the hormonal response is activated to some extent as part of stress reactivity (7). The first response is mediated *via* the sympathetic-adreno-medullar axis (SAM) i.e., increased sympathetic nervous system activation results in a so-called sympathetic “spillover” effect; the adrenal medulla responds to a high concentration of circulating norepinephrine by releasing epinephrine thus amplifying concentration of circulating catecholamines (8). At the same time, activation of the hypothalamic-pituitary (HP) axis results in the release of corticotropin-releasing factor (CRF), thyrotropin-releasing factor (TRH), and growth hormone-releasing factor (GHRH) from the hypothalamus, which stimulate the anterior pituitary gland to release specific hormones: adrenocorticotropic hormone (ACTH), thyroid-stimulating hormone (TSH), prolactin (PRL), follicle-stimulating hormone (FSH), luteinizing hormone (LH) and growth hormone (GH). As the exercise continues, other hormones from the anterior and posterior pituitary gland augment the responses of the SAM axis, as well as hormones from peripheral endocrine glands, such as cortisol, thyroxine (T4), triiodothyronine (T3), and their free forms (fT3 and fT4), as well as testosterone and insulin-like growth factor-1 (IGF-1) regulated by feedback loop mechanisms (9).

These hormones, together with the nervous system, control and coordinate the activity of all organ systems during exercise, including skeletal muscles. As a response, activated muscles become a source of numerous bioactive compounds, i.e., they express, produce, and secrete, various peptides and cytokines, classified as myokines (10, 11). Although recent studies identified over 600 myokines (12), the biological function of 95% of them remains undescribed and poorly understood (13). Since myokines exert autocrine, paracrine, and endocrine effects (14), they are responsible for crosstalk between the muscles and other organs including the brain, heart, adipose tissue, liver, pancreas, gut, bone, skin, and vascular system (14–18), having an impact on human health and ameliorating numerous diseases. One of the most interesting roles of myokines is in promoting the relationship between muscle and brain by mediating the muscle-brain endocrine loop (19, 20). In addition to interleukin 6 (IL-6), the first described and well-studied myokine, there are several myokines involved in the crosstalk between muscles and the brain, such as irisin, the N-terminal part of fibronectin type III domain-containing protein 5 (FNDC5) derived from the proteolytic cleavage in circulation (21); brain-derived neurotrophic factor (BDNF), a member of the neurotrophic factor family highly expressed in several brain regions, but also in muscles, and S100 calcium-binding protein B (S100 B), a member of S100 family, which is mainly found in astrocytes and Schwann cells, but also in some types of skeletal muscles (22).

However, available studies regarding the impact of rSCUBA diving on the neurohormonal system and the muscle-brain endocrine loop are scarce, but also very challenging to interpret due to the variety of studies' designs (e.g., depth, duration, type of breathing gas, and frequency of dives, anthropological proprieties, physical and overall health conditions of divers, their experience and level of training) and consequently, incomparability of data. In addition, some studies suggested possible adverse effects of diving on cognitive function, for which, at least partially, could be responsible a disturbed balance of crosstalk between muscle and brain, i.e., expression, secretion, and activity of hormones and myokines (23, 24).

To contribute to the understanding of adaptive mechanisms triggered by rSCUBA diving practiced once a week and get a more comprehensive insight into molecular events during and after the dive as well as its cumulative effects, we undertook a comprehensive longitudinal intervention study, which showed that rSCUBA diving repeated weekly triggers an adaptive response in cardiovascular (CV) and immune systems, muscles, and haematopoiesis (25–28). In this study, we aimed to explore neurohormonal events during and after rSCUBA diving, as well as, the cumulative effect of five dives practiced once a week, by measuring the serum concentration of ACTH, cortisol, TSH, free thyroxine (fT4), prolactin, total testosterone, GH, and insulin-like growth factor 1 (IGF-1), a primary mediator of the GH effects, whose main source is the liver but active muscles as well (29). To get a better insight into the crosstalk between muscles and the brain we measured the concentrations of irisin, BDNF, and S100B, but also neuron-specific enolase (NSE) and glial fibrillary acidic protein (GFAP), a sensitive neural cell body and glial cell injury markers, respectively, to rule out possible brain damage.

## Materials and methods

### Participants

This longitudinal intervention study included 14 male recreational divers, median age (range) of 42 (19–54) years, who had 3–20 years of diving experience and less than 30 dives per year. All divers had valid diving medical certificates, had not dived for at least five months (during the winter period), and were not professional athletes. Any acute or chronic illness symptoms were excluded by taking anamnestic data and laboratory tests before the beginning of this study. All participants were informed about the study's objectives and all of them signed consent to participate. The research was designed and carried out in accordance with the Helsinki Declaration and was approved by the Ethical Committee of the University of Zagreb Faculty of Pharmacy and Biochemistry.

### Study design

For assessing the cumulative effect of rSCUBA diving, five experimental dives (one dive per week) were conducted after a winter non-diving period, from March to April 2019 at the Adriatic seaside. The first dive was performed at a maximum depth of 20 m for a total of 30 min, and the remaining four dives at a maximum depth of 30 m for a total of 30 min. All five dives were carried out at the same time in the morning (between 9:00 am and 10:00 am), with gradual immersion to the maximum depth (descending velocity of 10 m/min) and gradual return to the surface (maximum ascending velocity of 9 m/min) without a decompression stop. Recommended safety stop was performed at a depth of 5 m and lasted 3 min at the end of each dive Diving equipment contained wetsuits, dive computers, and open-circuit SCUBA diving devices with compressed air as a breathing gas. The importance of the following diving protocol was explained to all subjects: diving in a group and adhering to the recommended velocity. Downloaded dive profiles from diving computers were used to analyse the depth and duration of the dive. The sea temperature was between 12 and 15°C and the air temperature was between 16 and 20°C.

### Blood sampling

From each subject, blood samples were collected immediately before (pre-dive) and after (post-dive) the first, third, and fifth dives (six samples per subject in total). Venous blood was drawn from the antecubital vein by trained laboratory professionals straight into vacuum tubes containing K2EDTA and clot activator. All collected samples were transported according to specific preanalytical requirements (in an ice bath) and delivered to the laboratory within 30 min.

Blood samples in test tubes containing K2EDTA, transported on the ice, were centrifuged immediately at 2,000 g for 10 min, and collected plasma samples were stored at −20°C for determination of ACTH. Blood samples in test tubes containing clot activator, transported in a cooled box, were centrifuged within 15 min at 2,000 g for 10 min, and collected serum samples were divided into 2 aliquots: (i) one (2 ml) was stored at −20°C for determination of TSH, fT4, prolactin, total testosterone, cortisol, GH, IGF-1, S100B, and NSE, and (ii) other (1 ml) stored at −80°C for determination of BDNF, irisin, and GFAP.

### Laboratory methods

ACTH plasma concentrations were determined by electrochemiluminescence immunoassay on Roche Cobas e411 analyser (Roche Diagnostics, Rotkreuz, Switzerland), as well as serum concentrations of GH, IGF-1, S100B, and NSE. Serum concentrations of TSH, fT4, prolactin, total testosterone, and cortisol were determined by the chemiluminescence microparticle immunoassay on Abbott Architect i2000SR (Abbott Diagnostics, Abbott Park, Illinois, United States). ELISA kits were used to determine serum concentrations of free BDNF (R&D Systems Inc., catalogue no. DBD00, Minneapolis, USA), irisin (BioVendor, catalogue no. RAG018R, Brno, Czech Republic), and GFAP (BioVendor, catalogue no. RD192072200R, Brno, Czech Republic) on the automated ELISA Siemens BEP 2000 Advance analyser (Siemens Healthcare Diagnostics Walpole, NJ, United States). All analyses were performed following the manufacturer's instructions.

### Statistical analysis

Statistical analysis was performed using Statistical Package for the Social Sciences (SPSS) (IBM SPSS Statistics 29.0.0, SPSS Inc., United States). All data were presented with median and interquartile range (IQR) based on the results of normality testing with the Kolmogorov–Smirnov test. Wilcoxon signed-rank test was used for testing the difference between pre- and post-dive results, while the Friedman test was used to assess the differences between all pre-dive results, as well as all post-dive results. When the Friedman test was positive (*P* < 0.05), *post hoc* analysis using Wilcoxon signed-rank tests was conducted with a Bonferroni correction applied, resulting in a significance level set at *P* < 0.017 (0.05/3 = 0.017).

## Results

Anthropometric data, expressed as median and IQR of 14 recreational divers were: height 1.80 (1.76–1.86) m, weight 85 (77–93) kg, and body mass index (BMI) 26.4 (23.5–28.5) kg/m^2^, respectively.

### Pituitary-organ axis hormones

Every single dive caused a statistically significant decrease in ACTH and cortisol post-dive concentrations when compared to the corresponding pre-dive values, while total testosterone post-dive concentration decreased statistically significantly only after the first dive (Table 1). No significant changes in post-dive concentrations of prolactin, GH, TSH, and fT4 were observed as compared to pre-dive values (Table 1). A Friedman analysis of pre- and post-dive results showed no statistically significant changes in any of the analysed parameters (Table 1).

**Table 1:** Results of pituitary-target organ axis hormones levels before and after the first, third, and fifth dive.

Parameter (unit)	DIVE 1		DIVE 3		DIVE 5		*P* (1 vs. 3 vs. 5)	
	Pre-dive	Post-dive	Pre-dive	Post-dive	Pre-dive	Post-dive	Pre-dive	Post-dive
ACTH (pmol/L)	6.9 (4.5–8.6)	3.4 (2.2–4.5)	6.1 (3.4–9.6)	4.1 (2.8–5.7)	5.3 (4.0–7.9)	3.6 (2.5–4.6)	0.109	0.135
*P* (post vs. pre)	↓ 0.001*		↓ 0.004*		↓ 0.002*			
Cortisol (nmol/L)	295.8 (244.9–350.3)	223.0 (189.1–270.9)	307.7 (256.2–355.6)	199.3 (174.6–300.4)	276.1 (217.5–389.0)	213.3 (163.8–338.0)	0.807	0.807
*P* (post vs. pre)	↓ 0.006*		↓ 0.019*		↓ 0.022*			
TSH (mIU/L)	1.09 (0.67–1.92)	1.33 (0.77–1.92)	1.11 (0.70–1.88)	1.45 (0.97–2.08)	1.04 (0.71–1.74)	1.27 (0.85–1.78)	0.500	0.926
*P* (post vs. pre)	0.133		0.055		0.133			
fT4 (pmol/L)	12.1 (11.5–12.9)	12.3 (11.7–13.0)	12.7 (11.4–13.4)	12.6 (11.4–13.2)	12.2 (11.4–13.1)	12.4 (11.5–12.8)	0.199	0.735
*P* (post vs. pre)	0.402		0.272		0.972			
Prolactin (mIU/L)	135.4 (112.5–187.3)	128.0 (98.3–167.3)	139.0 (101.6–198.2)	163.5 (102.5–229.4)	142.3 (124.1–196.4)	155.4 (99.9–220.2)	0.109	0.071
*P* (post vs. pre)	0.124		0.272		0.683			
Testosterone (nmol/L)	15.7 (14.0–21.0)	13.6 (12.5–17.6)	16.1 (13.9–22.8)	15.6 (13.5–19.0)	17.4 (15.7–20.4)	16.5 (14.6–18.9)	0.607	0.607
*P* (post vs. pre)	↓ < 0.001*		0.245		0.074			
GH (mIU/L)	0.55 (0.28–5.59)	1.5 (0.34–3.83)	0.44 (0.17–4.25)	1.64 (0.54–5.20)	0.69 (0.20–1.56)	0.64 (0.16–2.42)	0.410	0.265
*P* (post vs. pre)	0.826		0.551		0.552			

ACTH, adrenocorticotropic hormone; TSH, thyroid-stimulating hormone; fT4, free thyroxine; GH, growth hormone.

↓ A statistically significant decrease.

* A statistically significant difference between pre- and post-dive (Wilcoxon test).

### Myokines

**Figure 1:**
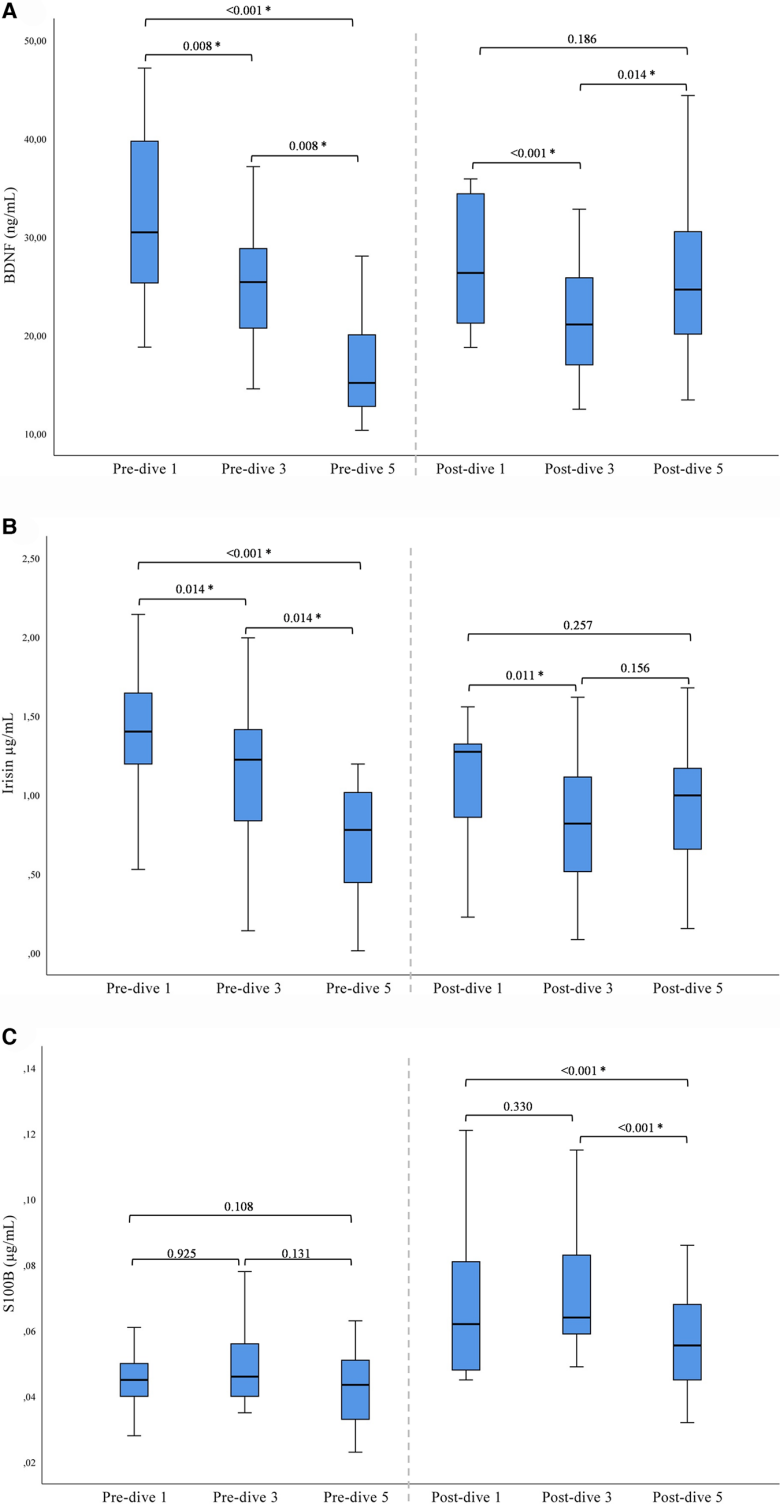
Results of post-hoc analysis for BDNF (**A**), irisin (**B**), and S100B (**C**) presented with box plots (median ± IQR). *Statistically significant change (*P* < 0.017) (Wilcoxon tests with a Bonferroni correction).

A statistically significant decrease in BDNF and irisin concentrations was observed after the first and third dives when post-dive values were compared to the corresponding pre-dive values. On the contrary, the fifth dive caused a statistically significant increase in BDNF and irisin post-dive concentrations as compared to the pre-dive values (Table 2). The cumulative effect of rSCUBA diving was reflected in a continuous decline in irisin and BDNF pre-dive levels throughout the whole studied period (Table 2 and Figure 1). For BDNP post-dive value, a statistically significant decrease was observed when comparing the third and first week, while a statistically significant increase was observed when the results obtained for the fifth and third weeks were compared. A comparison of the fifth and first weeks did not show significant changes (Figure 1). For irisin post-dive values, the same change trend was observed, although a statistically significant difference was not detected when comparing post-dive values between the fifth and third week. For S100B, a statistically significant increase was observed after each dive when compared to the pre-dive values (Table 2). The IGF-1 post-dive levels did not change significantly as compared to the corresponding pre-dive values (Table 2). Five dives practiced once a week had no cumulative effect on S100B and IGF-1 pre- or post-dive levels (Table 2).

**Table 2:** Results of myokines levels before and after the first, third, and fifth dive.

Parameter (unit)	DIVE 1		DIVE 3		DIVE 5		*P* (1 vs. 3 vs. 5)	
	Pre-dive	Post-dive	Pre-dive	Post-dive	Pre-dive	Post-dive	Pre-dive	Post-dive
BDNF (ng/ml)	30.48 (24.98–39.91)	26.34 (21.10–34.56)	25.41 (19.87–30.50)	21.10 (16.81–26.66)	15.16 (12.71–20.64)	24.66 (20.05–30.97)	< 0.001^#^	< 0.001^#^
*P* (post vs. pre)	↓ < 0.001*		↓ < 0.001*		↑ < 0.001*			
Irisin (µg/ml)	1.401 (1.172–1.647)	1.273 (0.814–1.326)	1.223 (0.733–1.444)	0.819 (0.455–1.114)	0.779 (0.444–1.020)	0.998 (0.648–1.198)	< 0.001^#^	0.036^#^
*P* (post vs. pre)	↓ < 0.001*		↓ < 0.001*		↑ 0.004*			
IGF-1 (nmol/L)	22.4 (17.3–26.2)	22.6 (18.9–26.0)	21.6 (17.7–28.1)	22.6 (17.7–28.6)	22.9 (19.1–26.4)	22.7 (19.5–26.2)	0.807	0.807
*P* (post vs. pre)	0.074		0.109		0.331			
S100*β* (µg/ml)	0.046 (0.039–0.053)	0.062 (048–0.084)	0.048 (0.040–0.060)	0.064 (0.059–0.085)	0.044 (0.032–0.052)	0.056 (0.044–0.070)	0.162	< 0.001^#^
*P* (post vs. pre)	↑ < 0.001*		↑ 0.023*		↑ < 0.001*			

BDNF, brain-derived neurotrophic factor; IGF-1, insulin-like growth factor 1; S100B, S100 calcium-binding protein B.

↓ A statistically significant decrease.

↑ A statistically significant increase.

* A statistically significant difference (*P* < 0.05) (Wilcoxon test).

^#^ A statistically significant difference (*P* < 0.05) (Friedman test).

### GFAP and NSE

GFAP post-dive levels decreased statistically significantly after every dive as compared to the baseline values (all *P* < 0.001), while NSE post-dive levels showed no significant change compared to the pre-dive values (Table 3). The cumulative effect of five dives practiced once a week was manifested in significant decreases in NSE pre-dive levels comparing the fifth to the first dive (*P* = 0.001) and the third dive (*P* = 0.014). In parallel, no cumulative effect on GFAP pre- and post-dive values was observed (Table 3).

**Table 3:** Results of GFAP and NSE levels before and after the first, third, and fifth dive.

Parameter (unit)	DIVE 1		DIVE 3		DIVE 5		*P* (1 vs. 3 vs. 5)	
	Pre-dive	Post-dive	Pre-dive	Post-dive	Pre-dive	Post-dive	Pre-dive	Post-dive
GFAP (ng/ml)	4.85 (4.51–5.16)	4.30 (4.05–4.69)	4.73 (4.59–4.89)	4.30 (4.13–4.48)	4.61 (4.51–5.00)	4.30 (4.05–4.40)	0.557	0.721
*P* (post vs. pre)	↓ < 0.001*		↓ < 0.001*		↓ < 0.001*			
NSE (ng/ml)	11.56 (9.67–14.12)	12.28 (9.24–14.89)	11.71 (9.12–12.90)	12.02 (11.18–14.77)	9.73 (8.16–11.61)	10.55 (9.02–11.71)	0.004^#^	0.395
*P* (post vs. pre)	0.730		0.074		0.363			

GFAP, glial fibrillary acidic protein; NSE, neuron-specific enolase.

↓ A statistically significant decrease.

*A statistically significant difference between pre- and post-dive (*P* < 0.05) (Wilcoxon test).

^#^ A statistically significant difference between Dive 1, Dive 3, and Dive 5 (*P* < 0.05) (Friedman test).

## Discussion

The presented results are part of a longitudinal intervention study that included 14 recreational SCUBA divers who performed five dives, one per week, on a depth of 20–30 m that lasted 30 min, after a non-dive period of 5 months. Blood samples were collected at six-time points: before and after the first, third, and fifth dives, in which the concentrations of selected endocrine hormones, myokines, and brain damage markers were measured.

Considering that diving with compressed air is a physical activity in extreme environmental conditions one could assume that due to the activation of SAM and HPA, rSCUBA diving results in increased levels of stress hormones. Yet, previous studies on rSCUBA diving effects on ACTH and cortisol neither confirmed nor reject that assumption, i.e., some studies observed increased cortisol (30, 31) and ACTH (30), levels, while in others no changes were observed (32, 33). Studies on the impact of rSCUBA diving on salivary cortisol are also controversial. For example, its increased levels were found after only one recreational dive to a depth of 10 m (23) whereas a recreational dive to 8 m resulted in a decrease in the salivary cortisol concentration (34). Our study showed a statistically significant decrease in the cortisol and ACTH levels after every dive as compared to the pre-dive levels, but no changes in their pre- or post-dive levels were observed during the studied period of one month. Similar results for cortisol were obtained in a study in which professional divers were exposed to moderate hyperbaria and normo- or hyperbaric oxygen (2.5 atm abs for 60 min) (33, 35). This discrepancy between studies results could be partly explained by different study designs and the level of expertise in SCUBA diving of the divers. Although the subjects included in our study were not professional divers it seems that their diving experience and positive attitude overcame physiological response to physical exercise, which commonly leads to an increase in cortisol (36). Diving is often preferred because it is an activity during which divers experience relaxation and stress reduction (37) while deep and controlled breathing leads to calmness, like meditation (38–40). In addition, this effect could be also contributed by a feeling of weightlessness, which is a result of the abolished effect of gravity due to buoyancy (41). However, it should be also taken into consideration that cortisol and ACTH are known for their distinctive circadian rhythm (42), i.e., they reach their peak concentrations between 6:00 and 10:00 am and significantly decline afterward. Since all pre-dive samples were collected between 8:45 and 9:00 am, while post-dive samples were collected between 10:00 and 10:30 am, it is possible that the observed decrease is additionally emphasized due to circadian changes. In any case, it seems that in our study design, rSCUBA diving did not activate the HPA axis and did not represent psychological stress for the divers.

We did not observe any statistically significant changes in prolactin levels after every dive, whereas a drop in total testosterone levels was observed after every dive, but a statistically significant decrease was observed only after the first dive. No cumulative effect in prolactin and testosterone levels was observed during the studied period of one month. Previous studies noticed a marked increase in plasma prolactin concentration after recreational dive to a depth of 8 m (34) and in the serum of recreational divers who performed physical work during the dive to a depth of 30 m (20 min) (43). The same study found a decrease in testosterone levels compared to the control group. These findings are at odds with a general belief that physical exercise leads to an increase in serum testosterone levels. Yet, numerous factors such as different types and intensities of exercise, recovery period duration, environmental conditions, and sampling time, significantly affect testosterone concentration (44). Furthermore, it is possible that the downward trend of testosterone levels observed in our study the trend could be a consequence of exposure to cold-water stress (45), as previously described in a group of trained male and female swimmers (46) but also in male cross-country skiers (47). Namely, it is possible that we observed the most pronounced decrease after the first dive because the sea and air temperatures at that time were the lowest during this study. Furthermore, it was the first dive performed after a non-dive period of 5 months, so it likely represented the biggest challenge for the organism, which afterward adapted to the challenging environment, so the observed changes were not so pronounced. However, it should not be disregarded that a decrease in testosterone levels triggered by rSCUBA diving might be also a consequence of acute exposure to the elevated environmental pressure on testicular perfusion as previously discussed by Verratti and collaborators (43), but the proposed mechanism needs to be studied further.

Physical activities in extreme environmental conditions, such as SCUBA diving affect the homeostasis of the organism, the regulation of which involves the hypothalamus-pituitary axis and consequently hypothalamic–pituitary–thyroid axis (HPT). Our study observed neither any statistically significant changes in TSF and fT4 levels after the dives nor over the studied period. Although thyroid hormones are involved in the normal functioning of skeletal muscles and the cardiovascular and pulmonary systems whose activity is significantly altered during the exercise, it is not yet clear if and how acute physical exercise affects TSH and downstream hormones. However, a recent study on a large cohort of healthy individuals observed no changes in TSH and fT4 levels after exercise (48).

No changes in growth hormone levels were observed, as well, neither after the dives nor during the studied period. The current understanding of the effects of physical exercise on GH levels is vague, due to the high complexity of the GH system, its sophisticated synthesis and processing that results in more than 100 GH isoforms existing in circulation, and usual differences that prevent comparison of such studies e.g., anthropometric data, lifestyle as well as a type, intensity, and duration of exercise. Nevertheless, an additional challenge represents a choice of GH assay and the large difference it can make in interpreting experimental data due to differences between bioactive GH and immunoreactive GH (detected by immunoassays) (49). We used a standard ELISA that measures immunoreactive GH, thus there is a space for speculation about the impact of weekly practiced rSCUBA diving on bioactive GH levels, and therefore further studies should be undertaken to clarify this question.

A part of the physiological effects of GH is achieved through insulin-like growth factor 1 (IGF-1), which together with IGF-2, six insulin-like growth factor-binding proteins (IGFBPs), and two insulin-like growth factor receptors, belongs to the IGF system (50). The liver plays a central role in the production of endocrine IGF-1, which is under the direct control of GH. But IGF-1 is also produced locally in many cells and tissues where it exhibits autocrine and paracrine effects. In myocytes, it is responsible for metabolic, mitogenic, and anabolic cellular responses (51, 52), and in the brain, it affects neuronal myelinisation and axonal sprouting and repairing damage (49, 53). Yet, during high-intensity physical activity, skeletal muscles become the primary tissue source for systemic IGF-1 production (54). Since IGF-1 can cross the blood-brain barrier by active transport (55), physical exercise *via* IGF-1 may contribute to neurogenesis. Nevertheless, the biological potency (bioavailability and activity) of the IGF-1 circulating form is fine-tuned by its binding proteins (IGFBPs) (49). Several studies showed upregulation of locally produced IGF-1 after both acute and chronic exercises, but studies on circulating IGF-I found decreased, increased, or not changed values (56, 57). In our study, statistically significant changes in serum IGF-1 levels were not recorded after individual dives or during the studied period. It is reasonable to suggest that rSCUBA diving, as a moderate-intensity physical activity, does not provoke either systemic IGF-1 production in muscles or increased GH-mediated production in the liver, since no changes in GH levels were observed. Yet, it remains possible that the time frame of blood sampling after the dive precluded the detection of changes in GH and IGF-1 levels.

It is widely accepted that as a response to exercise or muscle shivering due to exposure to coldness, myocytes release an adipo-myokine irisin, the N-terminal part of fibronectin type III domain-containing protein 5 (FNDC5) derived from the proteolytic cleavage in circulation (21). FNDC5 expression is under the control of the peroxisome proliferator-activated receptor γ coactivator 1-α (PGC1α), a metabolism regulator, thus irisin secreted from myocytes promotes mitochondrial biogenesis (58), whereas, in white adipose tissue (WAT), it promotes browning response and thermogenesis (59). Several studies showed increased irisin levels after exercise, suggesting positive effects of exercise on cardiovascular, skeletal, and muscular systems (60, 61). However, some other studies observed a decrease in irisin levels after various types of physical activity (62–66) whereas some others reported no changes in irisin levels in different exercise programmes (67, 68). In addition to these controversial findings, *in vitro* study did not support the proposed role of exercise-related signalling pathways in irisin regulation in human skeletal muscle (69). Herein, we report a statistically significant decrease in the serum concentration of irisin after the first and third dives, whereas after the fifth dive a statistically significant increase was observed. It is worth mentioning that although statistically significant, the irisin levels decreased by approximately 20% compared to the pre-dive values, and in the fifth dive increased by approximately 40% compared to the pre-dive values. Yet, a continuous decline in irisin pre-dive levels over the studied period (D3 vs. D1, D5 vs. D1 and D3) was observed. A decrease in irisin levels was also observed in marathon runners immediately after the run and 7 days after the race (62). Furthermore, a 3-month training intervention in middle-aged men led to decreased irisin (63), as well as a 9-month training of elite-level tennis players at the middle and at the end of the season (64). Irisin has multiple roles in the organism, among which it promotes cardiac progenitor cell-induced myocardial repair after the injury (70), affects blood pressure and controls hypertension through modulating vasodilatation as well as enhances endothelial barrier function (71) and prevents microvascular leakage thus improving pulmonary function, decreasing lung oedema and injury, and suppressing inflammation (72). It is interesting to observe that cardiomyocytes produce more irisin than skeletal muscle does (73). Therefore, it is reasonable to assume that decrease in irisin levels in circulation immediately after the dive reflects its increased consumption by various organs and tissues due to the augmented needs. The dynamics of changes in irisin levels (a decrease after the first and third dives, and an increase after the fifth dive) could be ascribed to the adaptation of the organism to the dives, i.e., reduced needs for irisin in muscles after five dives practiced once a week, which in turn results in increased irisin levels in circulation, observed after the fifth dive. It could be also related to the sea and air temperature at the time of the dive since they were lower when the first and third dives were performed as compared to the fifth dive. Although Bubak and collaborators reported no influence of the temperature in which exercise takes place on plasma irisin level (74), another study suggested increased consumption of irisin due to cold exposure and consequently decreased serum levels (66). It would be interesting to explore whether further dives would lead to the additional reduction in serum irisin levels (the pre-dive values) despite reversed trend observed after the fifth dive or the observed increase represents a turn-point that reverses the trend and lead to the return of the irisin levels to the initial values observed at the beginning of the study. Taken together, it seems that different types, intensities, and durations of physical exercise as well as environmental conditions strongly affect irisin production and consumption in various tissues and organs, which reflect in its blood levels.

Irisin can cross the BBB and in the hippocampus induce expression of brain-derived neurotrophic factor (BDNF), thus initiating neuroprotective mechanisms (75, 76). Namely, BDNF is a member of the neurotrophic factor family that has a crucial role in neuronal differentiation and plasticity, preserving cell viability and function, and preventing neuronal degeneration during stress (77). It is highly expressed in the brain, particularly in hippocampal neurons, but also in various nonneuronal tissues, including skeletal muscle cells, where it acts as a metabolic enhancer (78). BDNF exerts its biological roles by binding to its receptors, tropomyosin-related kinase B (TrkB) and p75 neurotrophin receptor (p75NTR). Many studies showed that exercise augments BDNF levels in the brain, skeletal muscle, and plasma, thus linking exercise to the induced improvement of cognitive functions (79–82). Interestingly, studies on animals showed increased BDNF mRNA and protein expression in muscle after exercise, but without muscle-derived BDNF release into circulation (83), suggesting that muscles are not the source of increased circulating BDNF after exercise. Most studies reported a range of BDNF concentrations between 8 and 46 ng/ml, with an average of around 18–26 ng/ml for healthy Caucasians (84). In our study, the interquartile range was 12.71–39.91 ng/ml, suggesting that the values remained within the physiological range. Although statistically significant, a decrease in BDNF levels observed after the first and third dives was rather small since the post-dive values were approximately 15% lower than the pre-dive values, whereas after the fifth dive it increased for 60% compared to the pre-dive value. BDNF can cross the blood-brain barrier in both directions (85) and a high positive correlation between serum BDNF levels and cortical and hippocampal BDNF expression was reported (86). Therefore, we assume that the decrease in BDNF levels, observed after the first and third dives, is a consequence of its leaving the circulation and sequestration by other tissues and organs, including the brain due to the increased needs, possibly due to increased production of reactive oxygen species in the periphery (25). Namely, it is unlikely that diving triggered its active elimination since BDNF is stable in blood up to 60 min after *i.v.* injection (85). It was recently shown that BDNF acts on pancreatic-islet-expressed-TrkB to promote peripheral insulin secretion, thus contributing to normalising hypoglycaemia (87), so it is possible that circulating BDNF enters the pancreas and in this way contributes to maintaining normoglycemia during diving in which glucose consumption is increased due to physical overload and low environmental temperature. Yet, the fifth dive resulted in BDNF levels increase, so the post-dive value was 60% higher than the pre-dive value, which was the lowest recorded BDNF level over the studied period. It is possible that after the increased demands for BDNF at the periphery or in the brain during the first several dives after the non-dive period of 5 months, adaptive mechanisms sensed enhanced demands and led to the increased BDNF production in the brain and other tissues, so the fifth dive triggered its release into circulation. It is interesting to notice that the dynamics of irisin changes followed the changes in BDNF values, because, as it was previously mentioned, irisin is one of the main regulators of BDNF expression in the brain. It is possible that irisin levels' lowering during the study period is related to the decline in BDNF levels, similarly as an increase in the concentration of both myokines observed after the fifth dive. Yet, it is hard to offer any conclusion since we can discuss only circulating irisin and BDNF levels not knowing anything about their concentration in tissues and organs. As in the case of irisin, it would be interesting to investigate the effect of the prolonged regular once-a-week practice of rSCUBA diving on circulating BDNF, as well as to check what is happening after the fourth dive. In addition, one should not disregard the finding that one 20 min diving session resulted in a short-term decrease in cognitive function in the group of professional SCUBA divers (23). Many studies reported a correlation between circulating BDNF levels and cognitive functions in healthy subjects but also those with various mental diseases (88, 89). A very recent study in which a deep narcosis test was administrated *via* a dry dive test at 48 (meter sea water, msw) in a hyperbaric chamber to six well-trained divers showed that dopamine/BDNF loss underscores narcosis cognitive impairment in divers (90), but in that study, the initial BDNF values dropped for approximately 50%. Although decreases in BDNF levels after the first and third dives were mild and most probably a part of the physiological stress response, it would be worthful to explore if the observed changes in circulating BDNF levels are somehow related to divers' cognitive functions but also the levels of BDNF-releasing neurotransmitters, such as such as dopamine and glutamate.

Another non-exclusive muscle tissue myokine is S100 calcium-binding protein B (S100B) which belongs to the S100 family, which members are distributed in three groups depending on the place of regulatory activity (29). S100B protein has an important role in the regulation of numerous cellular processes such as cell cycle progression and differentiation, regulation of cellular calcium homeostasis, and enzyme activities (91). It is mainly found in astrocytes and Schwann cells, but also in some types of skeletal muscles (22). Since it is predominantly expressed in astrocytes, it is most commonly used as a biomarker of traumatic brain injury (92). Yet, satellite cells, skeletal muscle cell precursors, also have relatively high concentrations of S100B, thus suggesting a possible role of S100B in the regulation of cellular processes that lead to muscle regeneration (29). Increased S100B levels were observed in marathon runners (93), rSCUBA divers (94), and breath-hold divers (95), whereas no changes in serum S100B levels were observed in an animal model of diving (96), in rSCUBA divers (97), as well as in rSCUBA divers with neurological decompression sickness (98). Increased circulating S100B levels after intensive exercise was ascribed to crosstalk between muscles and the brain, although increased values can also indicate active neural distresses. Therefore, to distinguish the source of increased S100B concentrations, it is required to include measuring of more sensitive markers such as glial fibrillary acidic protein (GFAP) and neuron-specific enolase (NSE) in these studies, thus enabling the ruling out acute brain injury (99). We found a statistically significant increase in S100B levels after each dive, but no changes in the pre-dive values were observed. We assumed that S100B originated from activated myocytes because we previously found increased post-dive plasma concentrations of myoglobin and galectin-3 at all-time points and a lowering of pre-dive and post-dive values of both biomarkers over the studied period (27). However, to rule out the possibility that increased S100B concentration in circulation is a consequence of the brain injury caused by diving, the concentrations of two more specific and sensitive markers of acute brain injury were determined GFAP and NSE (99). A statistically significant decrease in GFAP levels was observed after each dive whereas the pre-dive values remained stable over the studied period. Given that GFAP is produced predominantly by astrocytes, the decrease in peripheral blood levels reflects the changes in the brain. It was previously shown that physical exercise is followed by decreased GFAP expression in the hippocampus (100) as well as in circulating GFAP levels (101). A recent study that used a model of saturation diving (hyperbaric exposure to 401 kPa for 36 h) to explore if increased hydrostatic pressure or higher than normal oxygen and nitrogen partial pressures infer neuronal stress and possibly be harmful to the CNS, showed no significant increases in GFAP levels (102). Furthermore, changes in NSE values were not found after the dives and a statistically significant decrease in NSE pre-dive levels over the studied period (D5 vs. D1 and D3) was observed. Observed changes in GFAP and NSE concentration supported the assumption that S100B is released from muscles acting as a myokine rather than from the central nervous system indicating traumatic brain injury. A similar conclusion was reached in a study of rSCUBA divers who performed four open-water no-decompression dives to 18 msw lasting 49 min on consecutive days during which they performed moderate-level exercise (94). Our findings that increased S100B originates from activated muscles, that most profound elevation occurred after the first dive, and that an overall decrease in S100B levels was observed after the fifth dive indicated activation of an adaptive response in skeletal muscle by regular once-a-week diving. These results corroborate our previous findings that regular repetition of rSCUBA diving leads to functional adaptation of the muscular system after the most intensive physiological stress triggered by the first dive after the winter non-diving period (27).

### Study limitations

To the best of our knowledge and based on available scientific literature, this is the first study on associations between subsequent rSCUBA diving and serum concentration of TSH, fT4, GH, IGF-1, irisin, BDNF, and GFAP; however, several limitations must be highlighted. We were not able to investigate the impact of gender on obtained results since our study did not include female participants. Though our study included the largest number of participants among all the studies so far involving recreational SCUBA divers, it did not enable the grouping of the divers by either age or diving experience per year for further analyses of these factors' effects. Further, since only one month period of time was included, a longer period of time with a greater number of dives or additional sampling after the final dive could reveal more information on the cumulative effect of rSCUBA diving on the blood concentration of the selected biomarkers. Finally, only a narrow group of myokines involved in muscle-brain communication was investigated, measuring additional myokines could contribute to the understanding of the endocrine response complexity.

## Conclusion

The obtained results point to the specificity of rSCUBA diving as a moderate physical activity in extreme environmental conditions regarding the production of stress hormones, ACTH, and cortisol, but also myokines irisin and BDNF. Although the obtained results do not provide a complete picture but rather open numerous questions on endocrine response to rSCUBA diving and communication between muscles and brain, we believe that our findings contribute to the elucidation of molecular events underlying functional adaptation of the human organism to the extreme environment in which diving occurs.

## Data Availability

The raw data supporting the conclusions of this article will be made available by the authors, without undue reservation.
